# Genetic Profiles Playing Opposite Roles of Pathogenesis in Schizophrenia and Glioma

**DOI:** 10.1155/2020/3656841

**Published:** 2020-05-28

**Authors:** Ya-Dan Wen, Zhi-Wei Xia, Dong-Jie Li, Quan Cheng, Qing Zhao, Hui Cao

**Affiliations:** ^1^Department of Psychiatry, The Second People's Hospital of Hunan Province, The Hospital of Hunan University of Chinese Medicine, Changsha, Hunan, China; ^2^Department of Clinical Pharmacology, Xiangya Hospital, Central South University, 87 Xiangya Rd., Changsha 410008, China; ^3^Institute of Clinical Pharmacology, Central South University, Hunan Key Laboratory of Pharmacogenetics, 110 Xiangya Rd., Changsha 410008, China; ^4^National Clinical Research Center for Geriatric Disorders, 87 Xiangya Rd., Changsha 410008, Hunan, China; ^5^Department of Neurology, Hunan Aerospace Hospital, Changsha, Hunan 410205, China; ^6^Engineering Research Center of Applied Technology of Pharmacogenetics, Ministry of Education, 110 Xiangya Rd., Changsha 410008, China; ^7^Department of Geriatric Urology, Xiangya International Medical Center, Xiangya Hospital, Central South University, Changsha 410008, China; ^8^Department of Neurosurgery, Xiangya Hospital, Central South University, Changsha, Hunan, China

## Abstract

**Background:**

Patients diagnosed with schizophrenia were found having lower risks to develop cancers, including glioma. Based on this epidemiology, we hypothesized that there were gene profiles playing opposite roles in pathogenesis of schizophrenia and glioma.

**Methods:**

Based on GEO datasets and TCGA, key genes of schizophrenia genes on the opposite development of glioma were screened by different expressed genes (DEGs) screening, weighted gene coexpression network analysis (WGCNA), disease-specific survival (DSS), and glioma grading and verified by gene set enrichment analysis (GSEA).

**Results:**

First, 612 DEGs were screened from schizophrenia and control brain samples. Second, 134 key genes more specific to schizophrenia were left by WGCNA, with 93 key genes having annotations in TCGA. Third, DSS of glioma helped to find 42 key gene expressions of schizophrenia oppositely associated with survival of glioma. Finally, 24 key genes showed opposite expression trends in schizophrenia and different glioma grading, i.e., the upregulated key genes in schizophrenia expressed increasingly in higher grade glioma, and vice versa. CAMK2D and MPC2 were taken as the examples and evaluated by GSEA, which indeed showed opposite trends in the same pathways of schizophrenia and glioma.

**Conclusion:**

This workflow of selecting novel targeted genes which may have opposite roles in pathogenesis of two diseases was firstly and innovatively generated by our team. Some filtered key genes were indeed found by their potential effects in several mechanism studies, indicating our process could be effective to generate novel targeted genes. These 24 key genes may provide potential directions for future biochemical and pharmacotherapeutic research studies.

## 1. Introduction

The incidence of cancers in patients with schizophrenia was proposed lower than that of general population, firstly raised by the Board of Control of the Commissioners in Lunacy for England and Wales in 1909 [1]. A latest meta-analysis recruited schizophrenia patients from 16 cohort studies found decreased overall cancer incidence (RR = 0.90, 95% confidence interval (CI) 0.81–0.99), especially in lung cancer, colorectal cancer, liver cancer, stomach cancer, and prostate cancer [2]. Interestingly, the overall incidence of cancer in patients with schizophrenia did not parallel their cancer risk factor exposures [3]. Noteworthy, many potential confounding factors, including sex, ethnicity, genetic background, environmental exposure, and antipsychotic medications, influenced that the cancer prevalence among schizophrenia patients did not decrease in all types of cancer [2]. Therefore, some factors specifically related to schizophrenia may influence the tumorigenesis.

Considering that schizophrenia was well known for its heritability and familial transmission, the genetic components of schizophrenia may weigh heavily in the development of cancers. Some studies revealed significantly decreased risks of cancers in persons with schizophrenia and their relatives, suggesting that the familiar/genetic factors contributing to schizophrenia may potentially inhibit tumorigenesis and lead to the better survival [4–6]. In cerebral cancers, Grinshpoon et al. [7] reported that the standardized incidence ratios (SIRs) of the cancers in brain sites were significantly lower than 1.0 among men with schizophrenia (SIR = 0.56, 95% CI 0.32–0.81), suggesting a decreased risk of cerebral carcinomas in this group of people [7].

Some epidemiological studies showed that persons with schizophrenia may less likely to suffer from glioma [7, 8]. Gao et al. reviewed several genes involved in pathogenesis of schizophrenia play opposite roles in the development of glioma, such as neural progenitor proliferation, neurite outgrowth, neuronal migration, synapse formation, neurogenesis, and synaptic transmission and consolidation [9]. Not only the epidemiological information, there were some antipsychotic agents, such as pimozide, trifluoperazine, and brexpiprazole sensitizing glioblastoma or glioma stem cells, partially indicating that schizophrenia and glioma may have crosstalk on pathological mechanisms [3, 10, 11]. Above information provides hints, and we hypothesize that key genes of schizophrenia were crosstalk and negatively associated with the development of glioma. However, limited studies directly unveiled the association between schizophrenia genes and the survival of glioma. Therefore, identification of key genes of crosstalk between schizophrenia and glioma will be helpful to guide more insightful investigations and excavate novel targets of biochemical and pharmacotherapy research of schizophrenia and glioma in the future.

In summary, our study using a special gene expression profiles, based on GEO datasets, TCGA, and CGGA, first time, directly found 24 key genes of schizophrenia genes on the opposite development of glioma through different expressed genes (DEGs) screening, weighted gene coexpression network analysis (WGCNA), disease-specific survival (DSS), and glioma grading, and they were verified by Gene Set Enrichment Analysis (GSEA). These identified key genes through this workflow help to determine novel therapeutic targets for the treatments of schizophrenia and glioma.

## 2. Materials and Methods

### 2.1. Analysis Overview

**Figure 1:**
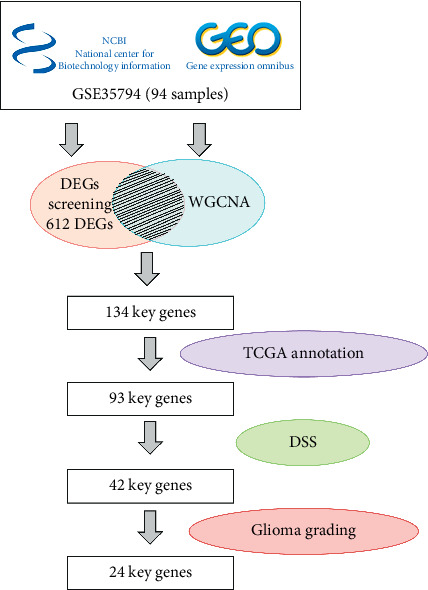
The workflow of screening the key genes playing opposite roles in schizophrenia and glioma.

In this study, the dataset about schizophrenia (SCZ) was downloaded and reanalyzed, which was GSE35794 (platform GPL6244) from the National Center of Biotechnology Information Gene Expression Omnibus, the most acknowledged gene expression resource for scientific community submitted data. All samples in this dataset were from cadaver with proper consent. The data were divided into two groups (the SCZ group and control group). DEGs between SCZs and control tissues were screened using the R software. Once DEGs were identified, functional and pathway enrichment analyses were used to analyze connections of DEGs and to determine the interaction of DEGs on the molecular level. Meanwhile, WGCNA was used to find the key genes positively and negatively related to schizophrenia. Then, DSS was used to find the key genes' opposite influence of survival in glioma and GETx and TCGA were used to evaluate the expression levels of these key genes in different glioma grading. Additionally, two genes were exhibited as examples and analyzed by GSEA to check whether the common pathways of schizophrenia and glioma in the two genes showed opposite trends or not. Lastly, six key genes were reevaluated in another brain tumor database, CGGA (Chinese Glioma Genome Atlas), through gene expression in different glioma grading and survival curves in patients with high- or low-level gene expression. The processing flow is shown in Figure 1.

### 2.2. Microarray Data Preprocessing and DEGs Screening

The raw data of GSE35794 were filtered out by probes with a corresponding gene symbol, and the average value of the gene symbols was calculated with multiple probes. Between the two groups, the Linear Models for Microarray Data Analysis (limma) package was used to screen the DEGs [12]. Threshold values were set as *p* < 0.05.

### 2.3. Functional Analysis of the DEGs

Nowadays, the most commonly trusted gene function knowledge bases are gene ontology (GO) and Kyoto Encyclopedia of Genes and Genomes (KEGG). In this study, we used a clusterProfiler package to analyze function profiles (gene ontology, GO; Kyoto Encyclopedia of Genes and Genomes, KEGG) of genes and gene clusters to identify major biological functions of genes [13]. All DEGs went through KEGG pathway analyses and GO analyses including the biological process (BP) using clusterProfiler.

### 2.4. Gene Network Construction and Module Detection

WGCNA was used to identify modules of coexpressed genes within gene expression networks [14]. To construct the network, the absolute values of Pearson correlation coefficients were calculated for all possible gene pairs. Values were entered into a matrix, and the data were transformed so that the matrix followed an approximate scale-free topology. A dynamic tree cut algorithm was used to detect network modules. WGCNA R package was used to evaluate the correlation of schizophrenia and module membership by the ‘p. weighted' function [15].

### 2.5. Key Genes of Schizophrenia Evaluated by DSS and Glioma Grading

After the DEGs were evaluated by WGCNA, the left genes were tested by DSS from TCGA. The upregulated key genes were intersected with hazard ratio (HR) < 1 of glioma (generated from DSS in TCGA, *p* value < 0.05), and downregulated key genes were intersected with HR > 1 of glioma (generated from DSS in TCGA, *p* value < 0.05) to find out key genes that may play opposite roles in schizophrenia and glioma.

Following that, the left key genes were evaluated by TCGA and the Genotype-Tissue Expression (GTEx) databases to observe the gene expression in different glioma grading. Expression data of glioma and normal controls were obtained from TCGA and the Genotype-Tissue Expression (GTEx) databases.

### 2.6. Gene Set Enrichment Analysis (GSEA)

GSEA helps to determine whether distinct sets of genes have significant differences using computational methods. We performed the GSEA analysis using the software clusterProfiler package of R language. Differences were considered statistically significant at |NES| > 1, nominal *p* value < 0.05, and FDR *q* value < 0.25. Then, the genes were used with the clusterProfiler package for analysis of the GO biological process. The cutoff value for the significant enrichment was set at *p* < 0.05.

### 2.7. Statistical Analysis

Statistical analyses and graphics were undertaken using R version 3.5.1. Student's *t*-test was used for the univariate analyses where appropriate. Survival rates of the expression level (high vs. low) were estimated by the Kaplan–Meier method with Rothman CIs. Survival curves were compared with the logrank test. The HR and 95% CI associated with the expressions of h-prune were estimated through a univariable Cox regression. A *p* value < 0.05 was considered statistically significant.

## 3. Results

### 3.1. Data Preprocessing and DEGs Screening

**Figure 2:**
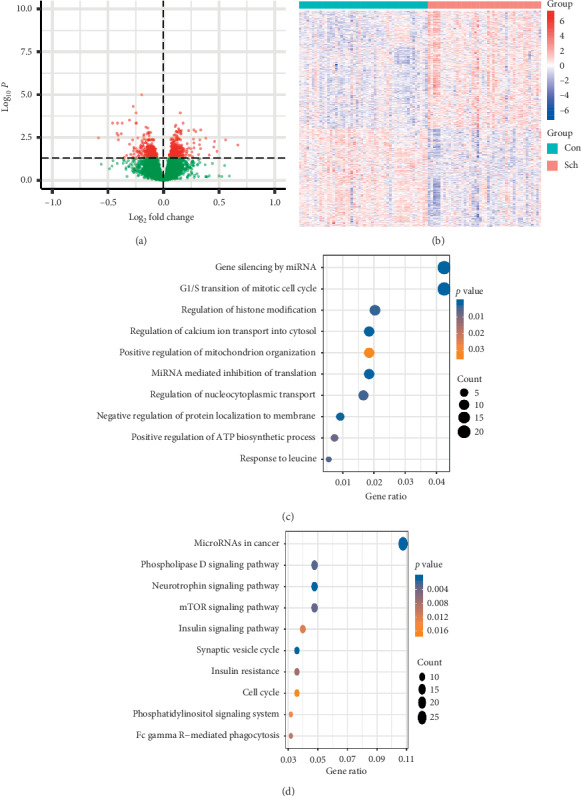
Functional Analysis of DEGs of schizophrenia. (a) Volcano plot for the DEGs. A total of 612 DEGs were screened out with a threshold of *p* < 0.05. (b) Heatmap showing the expression profiles of DEGs, with a gradual change in color from red to blue indicating high to low. (c) GO enrichment in molecular function with the 10 terms. (d) KEGG pathway enrichment analysis of common DEGs with 10 terms.

Removing the bipolar and depression samples, the dataset of GSE35974 contained 94 samples of the human cerebellum from schizophrenia and unaffected control in total 144 samples. The data of GSE35974 contained the clinical characteristics of age and gender, but not antipsychotic drug treatment. From the 94 samples, 612 DEGs were screened out with a threshold of *p* < 0.05. The limma package was employed to filter, and 332 upregulated genes and 280 downregulated genes were recognized afterward. A volcano plot and heatmap were depicted with the full picture of DEGs in all cases (Figures 2(a)–2(b)).

### 3.2. Functional Analysis of DEGs

By examining functions of DEGs, we had a better view about disease progression of schizophrenia. The GO analysis and KEGG pathway were employed to sort out DEGs. In GO biological processes, the most overrepresented are gene silencing by miRNA, G1/S transition of mitotic cell cycle, regulation of histone modification, regulation of calcium ion transport into cytosol, positive regulation of mitochondrion organization, miRNA mediated inhibition of translation, regulation of nucleocytoplasmic transport, negative regulation of protein localization to membrane, positive regulation of ATP biosynthetic process, and response to leucine (Figure 2(c)). In the KEGG pathway analysis, DEGs were notably enriched in MicroRNAs in cancer, phospholipase D signaling pathway, neurotrophin signaling pathway, mTOR signaling pathway, insulin signaling pathway, synaptic vesicle cycle, insulin resistance, cell cycle, phosphatidylinositol signaling system, and Fc gamma Rmediated phagocytosis (Figure 2(d)).

### 3.3. Schizophrenia Genes Screened by WGCNA

**Figure 3:**
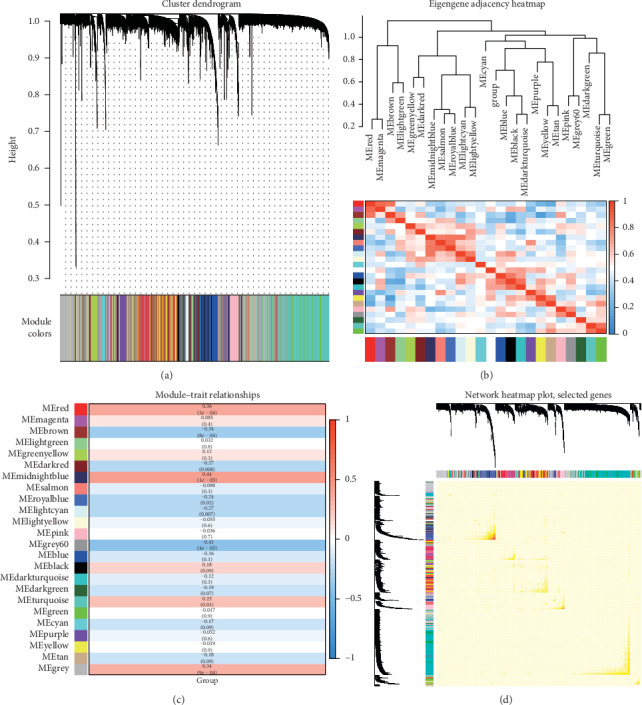
Modules features chosen by WGCNA. (a) Cluster dendrogram of modules identified by WGCNA. (b) Eigengene adjacency heatmap of module expression associations. (c) Module-trait relationships. (d) Network heatmap plot among selected genes.

Gene expression network analyses are an analyzing approach for describing the interactions among groups of transcripts so that the systematic alterations in expression could be observed. The modules identified by WGCNA were illustrated in a cluster dendrogram of modules identified by WGCNA, eigengene adjacency heatmap of module expression associations, module-trait relationship, and interesting genes in network heatmap (Figures 3(a)–3(d)), indicating that the clinical features were specific to schizophrenia. The module-trait relationship >0.3 was set as the modules positively related to schizophrenia, which were module midnight blue, red, and grey, and module-trait relationship <−0.3 was set as the modules negatively related to schizophrenia, which were module grey 60 and brown. Therefore, WGCNA was applied to evaluate gene expressions from SCZ and control samples in GSE 35974. The intersections of module midnight red with upregulated DEGs and the intersections of grey 60 with downregulated genes were zero. Finally, there were 26 upregulated key genes and 107 downregulated key genes identified by WGCNA, which found the genes close to clinical data of schizophrenia.

### 3.4. DEGs Screened by DSS in Glioma

**Figure 4:**
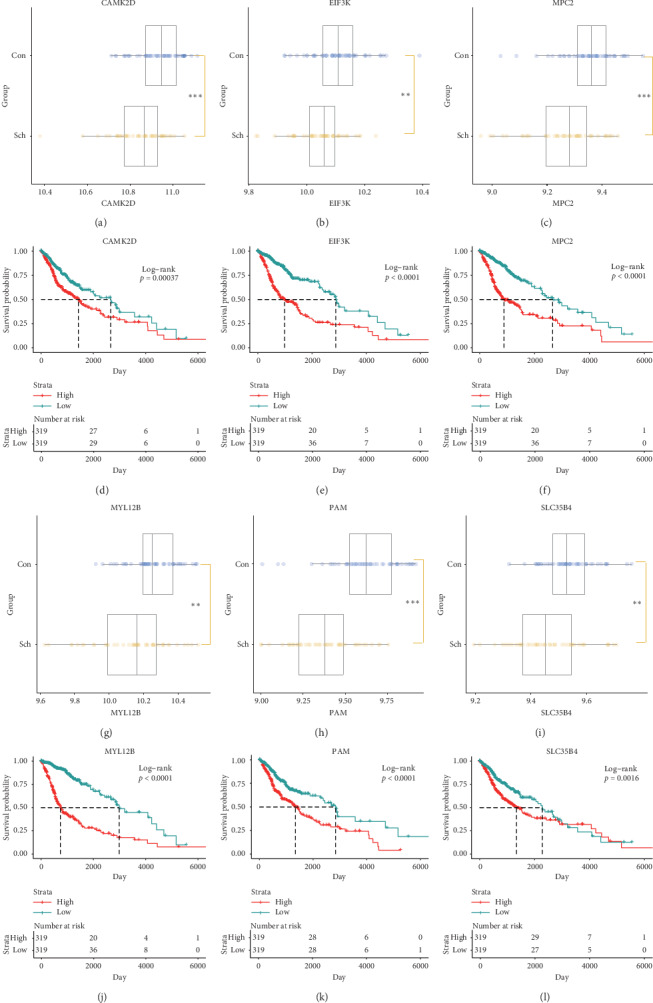
6 key genes as examples exhibiting the gene expression differences in schizophrenia and survival curves in glioma (TCGA). (a b, c g, h i) The differences of gene expression of CAMK2D (a), EIF3K (b), MPC2 (c), MYL12B (g), PAM (h), and SLC35B4 (i) in schizophrenia and control. (d e, f, j, k l) The survival curves of CAMK2D (d), EIF3K (e), MPC2 (f), MYL12B (j), PAM (k), and SLC35B4 (l) in glioma patients (TCGA). ^*∗∗*^*p* < 0.01, ^*∗∗∗*^*p* < 0.001.

Since schizophrenia patients had lower cancer incidence, the opposite gene expressions in glioma were expected to identify among 134 key genes filtered by WGCNA. DSS of glioma, a survival rate specific to glioma, was recognized as the evaluation method. Among 134 key genes, 93 key genes with annotations in TCGA were chosen for DSS. There were 6 upregulated key genes intersected with high risk in glioma and 36 downregulated key genes intersected with low risk in glioma through DSS, shown in Table 1. 6 most significant DEGs were chosen as examples to exhibit the difference between schizophrenia and control (Figures 4(a)–4(c) and 4(g)–4(i), ^*∗∗*^*p* < 0.01, ^*∗∗∗*^*p* < 0.001), and their survival curves of glioma (Figures 4(d)–4(f) and 4(j)–4(l)). Other DEGs are shown in Table 1 for your reference in your advance research.

### 3.5. Key Genes Evaluated by Glioma Grading

**Figure 5:**
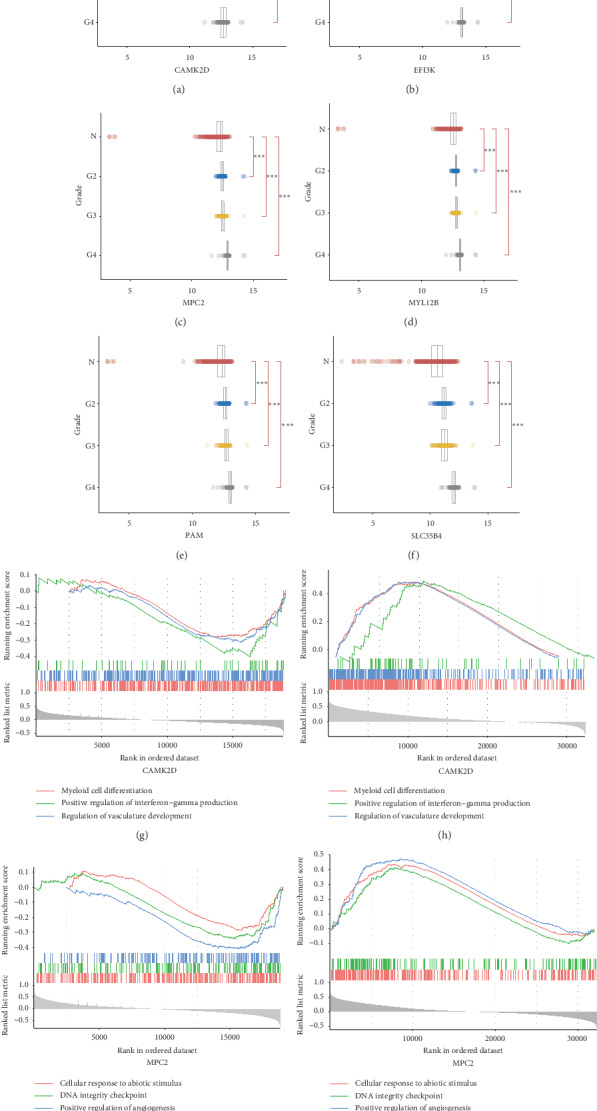
6 key genes as examples exhibiting their expressions in different glioma grading (TCGA) and 2 genes as examples to show their pathways opposite in schizophrenia and glioma (a–f) The trends of gene expressions of CAMK2D, EIF3K, MPC2, MYL12B, PAM, and SLC35B4 in different glioma grading (TCGA). (g–h) The GSEA analysis of CAMK2D in schizophrenia (g) and glioma (h). (i–j) The GSEA analysis of MPC2 in schizophrenia (i) and glioma (j). ^*∗∗*^*p* < 0.01, ^*∗∗∗*^*p* < 0.001.

Although 42 key genes were selected by WGCNA and DSS, the range of potential targeted genes for future biochemical and pharmacological research studies would be better to be compressed. Therefore, we considered that gliomas could be divided into low-grade gliomas (grade I and II) and high-grade gliomas (grade III and IV) according to the World Health Organization classification criteria. When the grade was higher (malignancy degree increased), the gene expression increased, meaning this gene may play a role in pathogenesis of glioma. Checking the expression levels of these genes in different glioma grading may help to observe the relationship of 42 key genes and the severity of glioma. If the expression level of a gene increases in higher-grade glioma, this gene may be closer to pathogenesis of glioma, and vice versa. Therefore, the upregulated key genes in schizophrenia were expected to find increased expression levels in higher-grade glioma, and downregulated key genes in schizophrenia decreased in lower-grade glioma. After observing the expression trends, 24 genes, i.e., ACOT9, ADA2, AP2M1, APMAP, APOO, ARPC2, CAMK2D, DHDDS, EIF3K, ERGIC3, EXTL2, FUNDC2, LZIC, MPC2, MYL12B, PAM, PRMT2, SLC35B4, TMEM167A, TMEM19, TSPAN13, VPS35, CNKSR2, and RTN4RL1, were accorded with the above expectation. 6 key genes (CAMK2D, EIF3K, MPC2, MYL12B, PAM, and SLC35B4) of schizophrenia are displayed as the expression trends in glioma grading in Figures 5(a)–5(f) (^*∗∗*^*p* < 0.01, ^*∗∗∗*^*p* < 0.001). CAMK2D and MPC2 were selected as the examples and evaluated by GSEA. In the common pathways of schizophrenia and glioma, these two genes showed opposite trends in schizophrenia and glioma, shown in Figures 5(j)–5(k).

**Figure 6:**
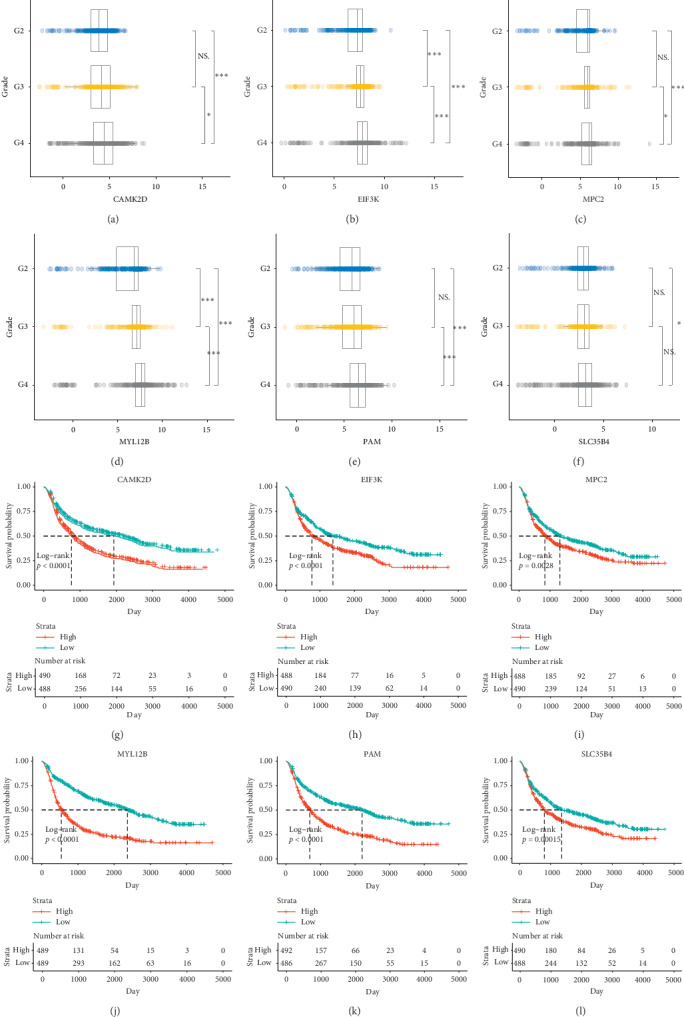
6 key genes, CAMK2D, EIF3K, MPC2, MYL12B, PAM, and SLC35B4, were reevaluated by CGGA in glioma grading and survival curves. (a–f) The trends of gene expressions of CAMK2D, EIF3K, MPC2, MYL12B, PAM, and SLC35B4 in different glioma grading (CGGA). (g–i) The survival curves of CAMK2D (g), EIF3K (h), MPC2 (i), MYL12B (j), PAM (k), and SLC35B4 (l) in glioma patients (CGGA). ^*∗∗*^*p* < 0.01, ^*∗∗∗*^*p* < 0.001.

CGGA, another brain tumor database, stored Chinese glioma datasets over 2,000 samples with mRNA sequencing, mRNA microarray, and matched clinical data to benefit the correlation and survival analysis. CAMK2D, EIF3K, MPC2, MYL12B, PAM, and SLC35B4, selected from the schizophrenia dataset and TCGA, were reevaluated in CGGA through gene expression in glioma grading and survival curves between patients having high level of gene expression and those having low level of gene expression. Luckily, the analyzed datum was quite in line with the above datum. These six key genes expressed increasingly in a higher glioma grade, shown in Figures 6(a)–6(f) (^*∗∗*^*p* < 0.01, ^*∗∗∗*^*p* < 0.001). Additionally, the survival rate was higher in patients with low gene expression level, shown in Figures 6(j)–6(k).

## 4. Discussion

Schizophrenia patients were found to have reduced overall risk of cancers compared to the general population, including the cancers of lung, melanoma, brain, breast, corpus uteri, and prostate. Additionally, there were several antipsychotic agents presenting their influence on glioma cells. Although the epidemiology and pharmacological evidences provided the association of schizophrenia and incidence of cancers, the gene profiles associated with underlying mechanisms between schizophrenia and glioma were still unclear. Gene profiling, based on our workflow, may provide the most related genes that play opposite roles in the two cerebral diseases.

In our study, the workflow first used DEGs screening, WGCNA, DSS, and expression in glioma grading to find the key genes that were significantly and differently expressed in schizophrenia patients, closely related to clinical data of schizophrenia, opposite influence of survival of glioma, and opposite trends of gene expressions in glioma, respectively. Finally, 24 key genes of schizophrenia were screened out, showing opposite influences in the survival of glioma and opposite gene expression trends in glioma grading, i.e., key genes upregulated in schizophrenia and low risk of glioma and key genes down-regulated in schizophrenia but also having high risk glioma. This workflow is an innovation of gene profiling to nicely find the intersection containing the key genes playing opposite roles in the two brain diseases.

Based on our findings, several proteins expressed by above genes were found in peers' mechanism studies both in schizophrenia and glioma. Interestingly, these genes indeed showed totally two direction effects, i.e., the risky genes in schizophrenia showed in glioma patients with good survival, while genes may prevent the development of schizophrenia showed in glioma patients with worse survival. Due to the limited studies, there were CAMK2D, EIF3K, MPC2, MYL12B, PAM, and SLC35B4 from 24 genes with references discussing their different roles in the two cerebral diseases.

CAMK2D encodes one of the subfamilies of calcium/calmodulin-dependent kinase II (CaMK II), which regulate Ca^2+^ homeostasis. In mammalian cells, CaMK II is composed of four different chains: *α*, *β*, *γ*, and *δ*. The encoded protein is contributed to this kinase *δ* chain [16]. Calcium/calmodulin-dependent kinase II alpha knockouts mice presented schizophrenia features that showed remarkable elevated levels of D2 receptors in their high-affinity state [17]. This trend was consistent with our results that CAMK2D expression was significantly lower in schizophrenia patients. Meanwhile, the activity of calcium/calmodulin-dependent protein kinase II was upregulated in resistant glioma cells and its cDNA transfection in sensitive glioma cells lead to glioma cells resistance, indicating that CaMK II may be involved in malignant glioma cell resistance [18]. This association was in line with our finding that CAMK2D expression was associated with the decreased DSS in glioma patients. Although there were some studies revealed the cell resistant by CaMK II may be through the Fas pathway, our findings provided a new vision of CAMK2D that may regulate common pathogenesis of schizophrenia and glioma so that the potential treatments of schizophrenia could be found in the glioma pathway, and vice versa.

MPC2, encoding one subunit of the mitochondrial pyruvate carrier (MPC) complex, a transporter protein in the mitochondrial inner membrane to control pyruvate transportation into the mitochondria, therefore plays a crucial role in the pathways of pyruvate metabolism and citric acid (TCA) cycle and glucose/energy metabolism [19]. The MPC complex contains MPC1 and MPC2, two obligate protein subunits. The loss in any subunit results in the destabilization of the MPC complex and thus dysfunction of the MPC complex [20]. Recent GWAS and meta-analysis in East Asian population showed MPC2 variant rs10489202 may be a risk locus for schizophrenia [21–23], indicating that expression of MPC2 may play a role in pathogenesis of schizophrenia. Additionally, abnormal MPC function was found in several cancers and contributed proliferation of cancers [20]. A glioma study found that MPC1 distinguished improved survival but MPC2 worsened survival in 1p19q-intact tumors (*p* < 0.01) [24]. These studies partially supported our finding that MPC2 expression was striking lower in schizophrenia patients and associated with deteriorated survival in glioma. Therefore, MPC2 could be a novel targeting gene to investigate new mechanisms between the two diseases.

EIF3K encodes eukaryotic translation initiation factor-3 (eIF3) subunit k, assembling with other 12 subunits to form the largest eIF3 complex, which implicates in mRNA translation initiation, termination, ribosomal recycling, and the stimulation of stop codon readthrough [25]. Recent studies found that the changes of expression of a single eIF3 subunit influence other subunit expressions [26], suggesting that changes of expression of any single eIF3 subunit may promote human disorders, including neurodegenerative disease, cancer, and infection. eIF3 was interacted with the protein encoded by a candidate gene of schizophrenia, disrupted-in-schizophrenia 1 (DISC1) gene, which was disrupted by a balanced chromosomal translocation, *t* (1; 11) (*q*42.1; *q*14.3) [27]. The translocation reduced DISC1 protein expression [28]. Overexpression of DISC1 promoted the assembly of the eIF3 complex [27], generating a hypothesis that lacks DISC1 protein may be hard to stimulate the expressions of eIF3 subunits. These studies not only agreed well our finding that the expression of EIF3K was decreased in schizophrenia but also supported to hypothesize that the protein encoded by EIF3K may be involved in schizophrenia pathogenesis. Additionally, the subunits eIF3 a, b, c, e, and f have been found as the oncogene overexpressed in several cancers, including nonsmall-cell lung cancer, breast cancer, cervical carcinoma, esophagus squamous-cell carcinoma, gastric carcinoma, and osteosarcoma. [25]. Recently, systematically profiling found that the expression of eIF3b, eIF3i, eIF3k, and eIF3m was increased with the glioma grade and poorer overall survival [29]. More studies showed knockdown of EIF3B [30], decreasing EIF3C [31], and silencing EIF3D [32] and EIF3E [33] alleviated proliferation and migration of glioma cells. Our profiling results were in accordance with above studies that increased expression of EIF3K was associated with decreased survival of glioma patients and increased glioma grades. Based on the peer investigations of pathogenesis on both schizophrenia and glioma, we have the reason to advise EIF3K as the potential targets of the mechanism study of schizophrenia and glioma, resulting new therapies for both diseases.

MYL12B encodes a subunit of myosin regulatory light chain 2 (MYL2), which regulates the activity of nonmuscle myosin II [34]. Knockdown of MYL12A/12B leads to dramatic changes of cell morphology and dynamics in NIH 3T3 cells [34]. A GWAS study found that the susceptibility genes of schizophrenia were associated with mRNA levels of MYL2 (*p* < 1.0*E* − 4) [35]. Another study conducted on postmortem brains from schizophrenia patients observed that MYL was phosphorylated in the anterior cingulate cortex [36]. Regarding to glioma, MYL was related to glioma cell migration [37], which can be blocked by inhibitors of myosin II [38]. Therefore, above studies of MYL and myosin II both on schizophrenia and glioma accorded with our findings that MYL12B was expressed lower in schizophrenia and was found decreasing DSS when glioma grade increasing. Although there were only several studies investigated on MYL12B, they could be the forerunner of exploring this potential intersecting gene, MYL12B, to excavate some novel minerals of the two diseases' mechanisms.

PAM encodes a preproprotein, peptidylglycine *α*-amidating monooxygenase (PAM), which is proteolyzed to generate the mature enzyme, including two distinct catalytic domains, a peptidylglycine *α*-hydroxylating monooxygenase (PHM) domain and a peptidylalphahydroxyglycine *α*-amidating lyase (PAL) domain [39]. These domains sequentially catalyze neuroendocrine peptides to active *α*-amidated products and regulate complex signaling between intestinal organs, peripheral neurons, and the central neuronal system [39]. PAM was recognized as one of the most promising candidate genes of schizophrenia [40]. Moreover, the expression of PAM was found increased in glioma cells [41]. There studies linked PAM to both schizophrenia and glioma, supporting our gene profiling that PAM may have a close relationship of both pathogenesis. Currently, there were limited mechanism studies of PAM on schizophrenia and glioma so that researchers may reveal deeper mechanisms that why an enzyme related to secreted peptides could conduct pathogenic or antipathogenic effects on the two diseases.

SLC35B4 encodes a subfamily of the solute carrier family of human nucleotide sugar transporters, which transport cytosolic nucleotide sugar to glycosyltransferases that reside in the lumen of the endoplasmic reticulum (ER) and/or Golgi apparatus [42]. Recently, SLC35B4 was evaluated as an empirically significant SNP of schizophrenia through a GWAS study [43]. In the cancer side, although there was no direct study of investigating the effects of SLC35B4 on glioma, several studies suppressed SLC35B4 expression to benefit the cancer therapies. SLC35B4 expression was markedly higher in gastric cancer tissues and involved in the progression of gastric cancer [44]. In advanced prostate cancer, a regulatory SNP, rs1646724, influenced SLC35B4 to promote the prostate cancer proliferation, migration, and invasion [45]. In glioma cells, SLC22A18 [46], SLC8A2 [47], SLC9A1 [48], and SLC34A2 [49] were examined as the risk genes of glioma. Our data also indicated that SLC35B4 may be involved in pathogenesis of both schizophrenia and glioma, which provide hints that researchers may shed light on this glycosyltransferase gene for drug development of both two diseases.

## 5. Conclusion

Through our process, 24 genes were sieved for future studies. Luckily, 6 genes were found by the mechanism studies both in schizophrenia and glioma. However, some biomedical investigations were not the direct indications. Additionally, the dataset of GSE35974 did not contain the clinical characteristics of antipsychotic drug treatment so that the influence of antipsychotic drug treatment could not be revealed. Therefore, further and deeper research could be conducted that why the two diseases shared gene profiles playing the opposite role in their pathogenesis. The negative overlap of risk genes between schizophrenia and glioma could hook more interests of investigators to discover novel pathways and potential pharmacotherapies based on our gene profiling.

## Figures and Tables

**Table 1:** 42 genes closely related to schizophrenia and glioma (TCGA) but may play opposite roles in the two diseases.

Upregulated key genes of schizophrenia intersected with high risk in glioma	CNKSR2, NPFFR1, RTN4RL1, WAPL, ZNF281, and ZNF519
Downregulated key genes of schizophrenia intersected with low risk in glioma	ACOT9, ADA2, AP2M1, APMAP, APOO, ARPC2, C19orf12, CAMK2D, CAP2, CFL1, CNR1, DHDDS, DYNLT3, EIF3K, ERGIC3, EXTL2, FDPSP2, FUNDC2, GPAT3, LAMTOR5P1, LZIC, MPC2, MRAP2, MYL12B, NRN1, PAM, PGK1, PRMT2, RHBG, SLC35B4, SNX10, TMEM159, TMEM167A, TMEM19, TSPAN13, and VPS35

## Data Availability

The datasets generated during and/or analyzed during the current study are available in the GO, TCGA, and CGGA repositories.
